# Risk of non-cardiac surgery after percutaneous coronary intervention with drug-eluting stents

**DOI:** 10.1038/s41598-017-16672-z

**Published:** 2017-11-27

**Authors:** Sun-Kyung Park, Dhong Eun Jung, Sung Ae Jung, Won Ho Kim, Jae-Hyon Bahk

**Affiliations:** Department of Anesthesiology and Pain Medicine, Seoul National University Hospital, Seoul, Republic of Korea

## Abstract

Elective non-cardiac surgery (NCS) should optimally be delayed one year after implantation of a drug-eluting stent (DES). Dual antiplatelet therapy or at least aspirin is recommended to be continued considering the relative risk of stent thrombosis especially during the 4 weeks after DES implantation. However, these recommendations were supported by insufficient evidence. We investigated predictors for postoperative major adverse cardiovascular and cerebral event (MACCE) in 1582 patients undergoing non-cardiac surgery after DES implantation. 96 patients (6.1%) developed postoperative MACCE. In the propensity score-matched analysis, aspirin maintenance was not associated with MACCE (odds ratio [OR] 0.78, 95% confidence interval [CI] 0.48–1.27, *P* = 0.320) and was associated with increased risk of major bleeding (OR 1.84, 95% CI 1.02–3.32, *P* = 0.044). When patients who underwent NCS within one month after DES implantation were matched with those who underwent NCS thereafter, the risk of MACCE was higher when surgery was done within 30 days after PCI (OR 2.21, 95% CI 1.05–4.66, *P* = 0.036). Maintenance of aspirin did not decrease MACCE after NCS in patients with DES and only increased the risk of major bleeding. NCS within one month after DES implantation was associated with higher incidence of MACCE. However, prospective trials are required to validate our results.

## Introduction

Perioperative management of the patients after previous percutaneous coronary intervention (PCI) with drug-eluting stent (DES) is challenging and has been an important topic of discussion^1^. Recent 2014 guidelines of European Society of Cardiologists (ESC)/European Society of Anaesthesiologists (ESA) and the 2016 focused update of 2014 guidelines of American College of Cardiology (ACC)/American Heart Associations (AHA) suggest that elective non-cardiac surgery (NCS) should optimally be delayed 6 months to one year after percutaneous coronary intervention (PCI) with drug-eluting stent (DES) due to risk of stent thrombosis^2–4^. Regarding the use of antiplatelet agents, dual antiplatelet therapy, or at least aspirin is recommended to be continued to prevent stent thrombosis especially during the first 4 to 6 weeks after DES implantation^3,5,6^.

Nonetheless, there is still substantial variability in the use of perioperative aspirin among patients undergoing noncardiac surgery, from those who are not yet taking aspirin to those who are already on long-term aspirin regimens. Regarding the antiplatelet therapy, a recent POISE-2 trial has shown that administration of aspirin before surgery and during the early postoperative period had no significant effect on the incidence of death or nonfatal myocardial infarction but only increased the risk of major bleeding^7^.

Recommendations on the interval from PCI to elective surgery are based upon several studies with different sample sizes and varying intervals between PCI and surgery^2^. A retrospective cohort study of more than 8,000 patients reported that the optimal time for elective surgery is >180 days after coronary stent insertion^5^. Another recent large retrospective cohort of more than 28,000 patients who underwent noncardiac surgery within 2 years of PCI reported that major adverse cardiac and cerebral events (MACCEs) were not associated with the stent type of both DES and bare metal stent (BMS) or surgical timing beyond 6 months after stent implantation^8^. After recent 2016 update of ACC/AHA guidelines, a retrospective cohort study of 4,303 patients with DES comparing with a matched control group without previous ischemic heart disease with similar surgeries reported that the risk of myocardial infarction increased only during the first month after DES implantation^9^. This study suggested that surgery after DES implantation may be performed earlier than 6 months without increased risk.

As such, the recommendations of previous guidelines are based upon conflicting and insufficient evidence about the risk of noncardiac surgery in patients with previous coronary stent insertion according to time from PCI to surgery and discontinuation of an antiplatelet agent. Therefore, the aim of our study was (1) to determine the independent risk factors for postoperative MACCE and the strength of their association, (2) to assess the incidences of postoperative MACCE according to time between PCI and surgery and (3) to compare the transfusion requirements and the incidence of major bleeding event according to different intervals from PCI to surgery and durations of antiplatelet agent administration prior to surgery. To achieve this aim, we undertook a retrospective cohort study of the patients who underwent noncardiac surgery after PCI with DES.

## Results

**Figure 1:**
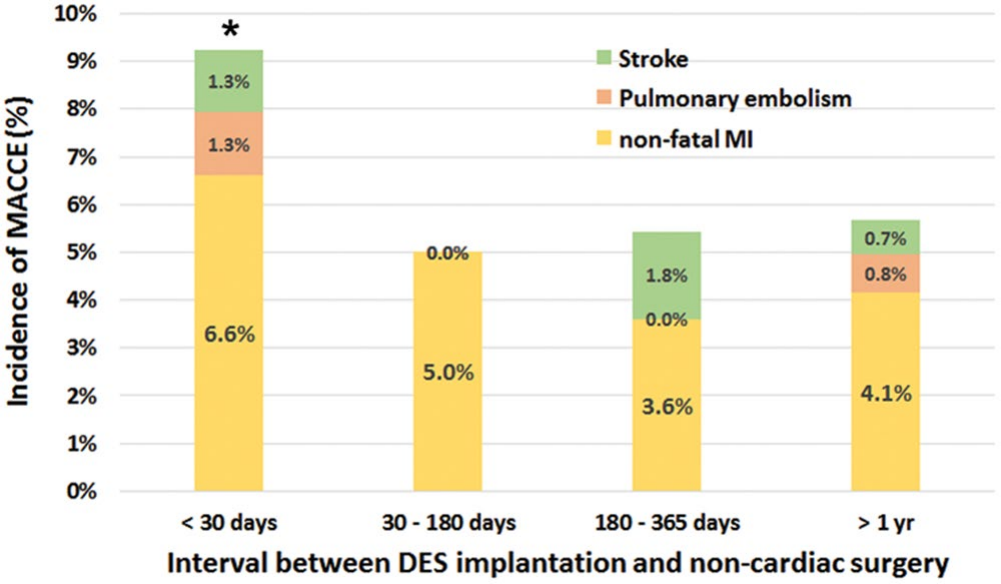
Frequency of postoperative MACCE in patients undergoging non-cardiac surgery after DES implantation according to the interval between DES implantation and surgery. The incidnece of MACCE <30 days after DES implantation was significantly higher than the incidences of the other intervals. MACCE = major adverse cardiovascular and cerebral event, DES = Drug eluting stent. *Incidence of MACCE was significantly higher than all the other intervals.

During the study period, a total of 1582 patients who underwent noncardiac surgery under general anesthesia within 5 years after PCI with DES were included in the analysis. The median (interquartile range) time from PCI to NCS was 608 days (143–1461). The overall incidence of postoperative MACCE was 6.1% (n = 96). The overall incidences of non-fatal myocardial infarction, pulmonary embolism, and stroke were 4.6% (n = 72), 0.7% (n = 11), and 0.8% (n = 13), respectively. However, we could not find any evidence of stent thrombosis in the patients who developed perioperative myocardial ischemia. The incidences of overall MACCE according to the different intervals between DES implantation and NCS were shown in Fig. 1.

Baseline patient characteristics according to the development of postoperative MACCE are shown in Table 1. The patients who developed postoperative MACCE were more likely to have chronic kidney disease, gone through vascular and musculoskeletal surgery, and had frequent intraoperative transfusion than those who did not develop postoperative MACCE. Our study population underwent high or intermediate risk surgery in the majority of cases^2^. The incidences of MACCE in patients who continued aspirin until surgery and those who stopped aspirin for >7 days or did not take aspirin prior to the operation were 6.9% (n = 31/452) and 6.1% (n = 39/636), respectively, without significant difference.

**Table 1:** Baseline patient characteristics by postoperative major adverse cardiovascular and cerebral event (MACCE).

Characteristic	Total (n = 1582)	Patients without postoperative MACCE (n = 1486)	Patients with postoperative MACCE (n = 96)	*P*-value
Demographic data				
Age, years, median [interquartile range]	70 (61–76)	70 (62–76)	68 (59–78)	0.392
Female, n	564 (35.7)	535 (36.0)	29 (30.2)	0.251
Body-mass index, kg m^−2^	23.4 (21.4–25.0)	23.4 (21.4–25.0)	23.8 (21.7–25.4)	0.206
Medical history				
Hypertension, n	974 (61.6)	916 (61.6)	58 (60.4)	0.811
Diabetes mellitus, n	612 (38.7)	567 (38.2)	45 (45.9)	0.089
Stroke, n	40 (2.5)	38 (2.6)	2 (2.1)	0.774
Chronic kidney disease, n	186 (11.8)	163 (11.0)	23 (24.0)	<0.001
Coronary stent type				
First generation DES	627 (39.6)	591 (39.8)	36 (37.5)	0.659
Sirolimus-eluting stent (Cypher)	285 (18.0)	268 (18.0)	17 (17.7)	0.936
Paclitaxel-eluting stent (Taxus)	356 (22.5)	336 (22.6)	20 (20.8)	0.686
Second generation DES (Xience, Endeavor, Resoult, Coroflex)	956 (60.4)	896 (60.3)	60 (62.5)	0.669
Time from PCI to surgery, days	608 (143–1461)	608 (153–1456)	580 (16–1690)	0.218
Time from PCI to surgery				0.184
<30 days	227 (14.3)	206 (13.9)	21 (21.9)	
30 days – 180 days	200 (12.6)	190 (12.8)	10 (10.4)	
180 days – 1 year	166 (10.5)	157 (10.6)	9 (9.4)	
>1 year	989 (62.5)	933 (62.8)	56 (58.3)	
Maintenance of aspirin until surgery without discontinuation, n	450 (28.4)	419 (28.2)	31 (32.3)	0.389
Maintenance of clopidogrel until surgery without discontinuation, n	219 (13.8)	206 (13.9)	13 (13.5)	0.930
Preoperative laboratory finding				
Hemoglobin, g dl^−1^	13 (11–14)	13 (12–14)	13 (11–14)	0.281
Albumin, g dl^−1^	4.2 (3.8–4.4)	4.2 (3.8–4.4)	4.1 (3.8–4.3)	0.061
Surgery-related parameter				
High-risk surgery				
Emergency surgery, n	40 (2.5)	36 (2.4)	4 (4.2)	0.300
Vascular surgery, n	129 (8.2)	112 (7.5)	17 (17.7)	<0.001
Intermediate-risk surgery				
Nose, mouth, and airway surgery, n	150 (9.5)	140 (9.4)	10 (10.4)	0.747
Digestive system, n	550 (34.8)	534 (35.9)	16 (16.7)	<0.001
Musculoskeletal surgery, n	217 (13.7)	194 (13.1)	23 (24.0)	0.003
Neurosurgery, n	59 (3.7)	55 (3.7)	4 (4.2)	0.779
Low-risk surgery				
Urologic surgery, n	200 (12.6)	192 (12.9)	8 (8.3)	0.190
Gynecologic surgery, n	42 (2.7)	42 (2.8)	—	
Miscellaneous, n	230 (14.5)	212 (14.3)	18 (6.1)	0.227
Colloid administration, n	0 (0–300)	0 (0–300)	0 (0–500)	0.052
Intraoperative red blood cells transfusion, n	105 (6.6)	93 (6.3)	12 (12.5)	0.017
Intraoperative fresh frozen plasma transfusion, n	89 (5.6)	81 (5.5)	8 (8.3)	0.248

Values are expressed as mean (SD), median (interquartile range) or number (%).

DES = drug-eluting stent; PCI = percutaneous coronary intervention.

Table 2 shows the results of univariable and multivariable logistic regression analyses for postoperative MACCE. History of chronic kidney disease, operation time, surgery type of vascular and musculoskeletal surgery, and intraoperative RBC transfusion were significantly associated with postoperative MACCE on univariable analysis. The doses of aspirin and clopidogrel administered before surgery were 100 mg day^−1^, and 75 mg day^−1^, respectively in all patients. Multivariable analysis identified that history of chronic kidney disease, operation time, vascular surgery, and musculoskeletal surgery were associated with postoperative MACCE (*P* < 0.005). Maintenance of aspirin until surgery without discontinuation was not protective against postoperative MACCE (maintenance of aspirin until surgery, adjusted odds ratio [OR] 1.10, 95% confidence interval [CI] 0.65 to 1.85, *P* = 0.717).

**Table 2:** Multivariable analysis of patient characteristics associated with postoperative MACCE.

Variable	Univariable Analysis		Multivariable Analysis	
	Odds Ratio (95% CI)	*P*-value	Odds Ratio (95% CI)	*P*-value
Age, year	1.01 (0.99–1.03)	0.476		
Female	0.79 (0.49–1.27)	0.331		
Body-mass index, kg m^−2^	1.03 (0.97–1.10)	0.321		
Hypertension	0.95 (0.62–1.45)	0.811		
Diabetes mellitus	1.43 (0.95–2.16)	0.091		
Chronic kidney disease	2.56 (1.56–4.20)	<0.001	2.28 (1.36–3.81)	0.002
Stroke history	0.81 (0.19–3.41)	0.775		
Coronary stent type, 2^nd^ generation vs. 1^st^ generation	1.10 (0.72–1.68)	0.669		
Operation time, min	1.03 (1.01–1.04)	<0.001	1.03 (1.01–1.04)	<0.001
Preoperative hemoglobin <10 g dl^−1^	1.62 (0.72–3.62)	0.244		
Preoperative albumin <3.3 g dl^−1^	0.99 (0.47–2.09)	0.979		
Emergency operation	1.75 (0.61–5.03)	0.298		
Vascular surgery	2.64 (1.51–4.61)	0.001	2.38 (1.31–4.32)	0.004
Musculoskeletal surgery	2.10 (1.28–3.43)	0.003	2.81 (1.68–4.72)	<0.001
Colloid administration, ml	1.00 (1.00–1.01)	0.097		
Intraoperative pRBC transfusion	2.14 (1.23–4.06)	0.020		
Maintenance of aspirin until surgery without discontinuation	1.22 (0.78–1.89)	0.389	1.10 (0.65–1.85)	0.717
Maintenance of clopidogrel until surgery without discontinuation	0.97 (0.53–1.78)	0.930	0.79 (0.39–1.61)	0.516

CI = confidence interval; pRBC = packed red blood cells; FFP = fresh frozen plasma, PCI = percutaneous coronary intervention.

Table 3 shows the results of propensity score-matched analysis. In the matched analysis of aspirin maintenance, the risk of MACCE was not different according to aspirin maintenance (OR 0.78, 95% CI 0.48 to 1.27, *P* = 0.320). However, the risk of major bleeding was significantly higher in patients who maintained aspirin than those who stopped aspirin (OR 1.84, 95% CI 1.02 to 3.32, *P* = 0.044). In the matched analysis of surgery intervals, the incidence of MACCE was significantly higher in surgery performed before one month after DES implantation than surgery after one month (OR 2.21, 95% CI 1.05 to 4.66, *P* = 0.036). The incidence of MACCE was not different in the matched cohort according to the cutoffs of 6 or 12 months after DES implantation.

**Table 3:** Results of propensity score matching analysis.

Matched Variable	Outcome Variable	*P*-value	Odds ratio (95% CI)	*P*-value
Maintenance of aspirin until surgery	MACCE (n)			
Maintenance (n=450)	31 (6.9)	0.319	0.78 (0.48 – 1.27)	0.320
No maintenance (n=450)	39 (8.7)			
Maintenance of aspirin until surgery	Major bleeding (n)			
Maintenance (n=450)	32 (7.1)	0.042	1.84 (1.02 – 3.32)	0.044
No maintenance (n=450)	18 (4.0)			
Surgery intervals	MACCE (n)			
Before 30 days (n=235)	23 (10.1)	0.032	2.21 (1.05 – 4.66)	0.036
After 30 days (n=235)	11 (4.8)			
Before 180 days (n=427)	31 (7.3)	0.407	1.26 (0.73 – 2.17)	0.408
After 180 days (n=427)	25 (5.9)			
Before one year (n=593)	40 (6.7)	0.471	1.19 (0.74 – 1.91)	0.472
After one year (n=593)	34 (5.7)			

MACCE = major adverse cardiovascular and cerebral event. No maintenance means aspirin use before surgery but stopped ≥7days before surgery or no use within 30 days before surgery.

Table 4 shows the comparison of the incidence of transfusion amounts and major bleeding according to the maintenance or discontinuation of aspirin or clopidogrel before surgery. The incidence of transfusion and major bleeding was significantly higher when aspirin was maintained before surgery.

**Table 4:** Comparison of the incidence of intraoperative transfusion requirements and major bleeding according to the maintenance or cessation of aspirin and clopidogrel before surgery.

Drugs use	Intraoperative red blood cell transfusion	Intraoperative Fresh frozen plasma transfusion	Major bleed
Maintenance of aspirin until surgery without discontinuation (n = 450)	40 (8.9)	34 (7.6)	32 (7.1)
No maintenance of aspirin (n = 1132)	65 (5.7)	55 (4.9)	30 (2.7)
*P*-value	0.023	0.040	<0.001
Maintenance of clopidogrel until surgery without discontinuation (n = 219)	19 (8.7)	15 (6.8)	13 (5.9)
No maintenance of clopidogreal (n = 1363)	86 (6.3)	74 (5.4)	49 (3.6)
*P*-value	0.192	0.397	0.097

The *P*-values of comparison of incidences were the results of chi-square tests. Data are presented as number (%).

## Discussion

Our retrospective study investigated the risk factors that predict the postoperative MACCE after noncardiac surgery including time from PCI to surgery and maintenance of antiplatelet agent in 1582 patients who underwent noncardiac surgery after PCI with DES. Maintenance of aspirin before surgery was not protective against the development of postoperative MACCE. The incidence of postoperative MACCE was significantly higher when surgery was performed within one month after PCI than thereafter. There was a higher incidence of transfusion during surgery when aspirin was maintained preoperatively and maintenance of aspirin was a significant predictor for major bleeding after multivariable adjustment. These findings were consistent in the propensity score analysis. However, our sample size was validated from the predicted odds ratio of the intervals from PCI to surgery for postoperative MACCE and multivariable analysis for the development of postoperative MACCE. The comparison of major bleeding and transfusion may be underpowered.

In 2014, ACC/AHA released updated guidelines on perioperative cardiovascular evaluation and management of patients having non-cardiac surgery^3^. These guidelines recommended that dual antiplatelet therapy should be continued in patients undergoing NCS during the first 4 to 6 weeks after BMS or DES implantation unless the risk of bleeding outweighs the benefit of stent thrombosis prevention^3^. This recommendation was due to the concern that early stent thrombosis can occur with both the bare metal stent and DES. However, guidelines did not refer to any previous study supporting this recommendation. ESC/ESA 2014 guidelines also recommended the continuation of aspirin in patients previously treated, and the decision should be based on weighing the risk of thrombotic complications and uncontrollable bleeding during surgery^2^. This recommendation was supported by several references including POISE-2 trial^7,10,11^.

There have been studies supporting the use of preoperative aspirin to decrease thrombotic complications^10,12,13^. In a previous study^12^, a total of 220 patients at high risk of the perioperative major adverse cardiovascular event (MACE) were randomized to take either 75 mg aspirin or placebo. Continuing aspirin during perioperative period significantly reduced MACE and did not increase perioperative bleeding-related complications. Other previous literature also suggested that the discontinuation of aspirin before surgery resulted in an increased thrombotic risk^10,13^.

On the other hand, there have been also studies that did not support the hypothesis that perioperative aspirin use may prevent postoperative MACE^14–16^. A previous randomized trial of 291 patients reported no difference in bleeding or thrombotic risks after elective non-cardiac surgery regardless of preoperative maintenance or cessation of aspirin^15^. The POISE-2 trial randomized 10,010 patients who were preparing to undergo noncardiac surgery and showed that aspirin did not reduce the risk of death or nonfatal myocardial infarction but only increased the risk of major bleeding^7^. However, the POISE-2 trial did not exclusively enroll patients with a previous DES but only enrolled patients at risk for vascular complications including those with a history of coronary artery disease. In addition, the findings of the POISE-2 trial were criticized because of methodological issues^17,18^. Based on the details of the POISE-2 trial, nearly two-thirds of subjects in the aspirin group received anticoagulant prophylaxis, the incidence of venous thromboembolism was very low, and out of all surgery types, only 6.1% was vascular surgery^17,18^. Thus, the true impact of aspirin on the high-risk patients may not have been evaluated fully. Furthermore, a systematic review of randomized trials including POISE-2 and pulmonary embolism prevention trial demonstrated that aspirin is still effective for the prevention of deep vein thrombosis and pulmonary embolism^18^.

Notably, a previous prospective trial attempted to find an association between preoperative platelet function test using a platelet mapping assay and postoperative MACE^16^. The study results could not demonstrate that MACE was associated with lack of platelet inhibition, suggesting that a substantial proportion of postoperative myocardial infarction was due to supply-demand mismatch from perioperative bleeding rather than stent thrombosis^19^. In this regard, maintenance of antiplatelet agent may only increase the risk of bleeding and may not be beneficial to prevent perioperative ischemia. Our study results also did not support the preoperative use of aspirin, as maintenance of aspirin until surgery was not associated with decreased postoperative MACCE. However, our results should be interpreted cautiously considering the retrospective design.

With respect to the timing of elective noncardiac surgery after DES implantation, both guidelines provided similar suggestions^3,5^. Elective noncardiac surgery should be delayed one year after DES implantation, although this delay may be reduced to 6 months for the new-generation DES^3,5,14,20–22^. However, two large retrospective observational studies reported that incidences of major adverse cardiac events (MACE) did not differ when surgery was postponed after 180 days from the DES implantation^5,8^. Based on these two studies, ACC/AHA 2014 guidelines and 2016 focused update commented that elective noncardiac surgery after DES implantation may be considered after 180 days^3,4^. Rossini and colleagues^23^ performed an analysis of multicenter, prospective registry of 1082 patients with previous coronary stent insertion. Surgeries performed within 180 days from PCI were associated with net-adverse clinical events, but not with MACE.

In our study results, the incidence of MACCE was significantly higher when surgery was performed within 30 days after PCI than other intervals. The incidence of MACCE did not differ thereafter. A recent large retrospective study reported a similar result^9^. They conducted a matched cohort study comparing patients who received DES with the patients who underwent similar surgeries without previous ischemic heart disease. The risk of myocardial infarction increased only during the first month after DES implantation. Although the types of surgery and patient characteristics may have influenced our study results, the risk of immediate surgery within one month following PCI with DES appears to be consistent across the previous literature^5,8,14,20,21^. However, prospective studies are required to support that surgery might be undertaken earlier than current guidelines.

The incidence of transfusion of RBC and FFP was significantly higher when aspirin was maintained preoperatively. Aspirin maintenance was associated with increased odds for major bleeding even after multivariable adjustments and propensity score analysis. Regarding the impact of aspirin on bleeding events after elective non-cardiac surgery, previously-mentioned prospective observational and randomized trials are notable^14,15^. Bleeding risks can vary significantly according to the time and dose of last taken aspirin, or bleeding risk of surgery. The validity of analyses of clopidogrel in our study is limited because more than half of patients underwent surgery more than one year after PCI and therefore only 219 patients (14%) were on clopidogrel before surgery among our study population.

The present study has several important limitations to address. Firstly, our study data were derived from a single-center, retrospective cohort. Postoperative MACCE and composite of morbidities were retrieved from electronic medical records, which may have resulted in an underestimation of true incidence. It is possible that some endpoints were misclassified. Secondly, we included patients who underwent NCS within 5 years after PCI and data were collected over 12 years. Over this long period, the standard of care for patients with DES and surgical procedure may have evolved, which may alter important covariates used to predict MACCE. Thirdly, more than half of the patients had the interval of more than 1 years since PCI and did not take the dual antiplatelet therapy at the time of the surgery. The role of dual antiplatelet therapy and clopidogrel could not be analyzed in our study. Fourthly, the composition of surgery type may influence our study results. Only 10.3% (164/1582) of our study population underwent high-risk vascular or emergency operation. The true impact of the time from PCI to surgery or the cessation of the antiplatelet agent may be diluted with moderate and low-risk surgeries. Sixthly, the definition of bleeding outcome may influence our results. The definition of major bleeding from the POISE-2 trial may not be a broadly accepted scale and using another scale may lead to different results^23,24^.

In conclusion, our single-center retrospective analysis showed that continuation of aspirin until surgery in patients who received previous DES was not associated with decreased incidence of MACCE after NCS and was associated with increased risk of major bleeding. Therefore, our study results do not support maintenance of aspirin before non-cardiac surgery in patients with previous DES. The surgery performed within one month after PCI with DES was associated with high risk of MACCE. However, our study was limited by the small sample size, retrospective design, and including less than half of patients who underwent surgery within a year from PCI. No definite recommendation can be made. Large clinical trials evaluating the effect of preoperative aspirin administration and interval from DES to surgery in patients undergoing NCS with DES are still required to give answers to these important questions.

## Methods

This retrospective cohort study was approved by Seoul National University Institutional Review Board (1604-023-753) and was registered at https://clinicaltrials.gov (NCT 02941588). Written informed consent was waived due to the retrospective nature of the present study. Electronic medical records of adult patients who underwent non-cardiac surgery within 5 years after percutaneous coronary intervention with a drug-eluting stent at Seoul National University Hospital between April 2004 and August 2017 were reviewed. Patients who received a sirolimus-eluting stent (Cypher), a paclitaxel-eluting stent (Taxus), an everolimus-eluting stent (Xience) or zotarolimus-eluting stent (Endeavor, Resolute) were included in the analysis. Patients who underwent general anesthesia after coronary angiography were initially reviewed. The patients were identified from electronic medical record database of our hospital according to the prescription codes of the national health insurance for coronary angiography and general anesthesia. From 4676 patients who met these criteria, the following patients were excluded; those who underwent cardiac surgery (n = 1357) and who did not receive PCI with DES at the time of coronary angiography (n = 1737). Resulting 1582 patients who underwent noncardiac surgery under general anesthesia within 5 years after PCI with DES were finally included in the analysis.

Baseline clinical parameters and demographic factors which were previously known to be related to postoperative morbidities and MACCE were included in this study after literature review (Table 1)^8,25^. The factors included medical history, preoperative medication, preoperative laboratory values, surgery-related factors and anesthetic details including transfusion-related parameters. The following data were obtained: time interval from PCI to surgery, coronary stent type, maintenance of antiplatelet therapy after PCI (none, aspirin, clopidogrel, or dual antiplatelet therapy), discontinuation of antiplatelet therapy before surgery (categorized as continued until surgery without discontinuation, used before surgery but stopped ≥7days before surgery, no use within 30 days before surgery). Patients who stopped aspirin or clopidogrel for 1 to 6 days were excluded from the analyses for the effect of maintenance or discontinuation of these drugs due to the possible residual effect of varying degree.

The primary endpoint was the major adverse cardiovascular and cerebral event (MACCE), which was defined as a composite of non-fatal myocardial infarction, coronary revascularization, pulmonary embolism and stroke within postoperative 30 days and was retrospectively collected by screening medical charts. The incidence of outcome after patient discharge from hospital was investigated by reviewing the outpatient chart. The secondary endpoint was a composite of overall postoperative morbidities, including MACCE, respiratory, cardiac, renal and other complications within postoperative 30 days. Definitions of individual components of MACCE and morbidity are described in Supplementary Tables S1 and S2.

The influence of discontinuation pattern of aspirin and clopidogrel on bleeding was evaluated by comparing the incidence of major bleeding and transfusion requirements during surgery. A major bleeding event was defined according to a previous study as follows:^7^ (1) Transfusion of ≥2 units of RBC during the surgery with a preoperative hematocrit ≤30% or a drop of hematocrit ≥10% from baseline, or (2) Requiring transfusion of ≥4 units of red blood cells within a 24 hour period, or (3) Requiring any one of the following interventions (i.e., embolization, superficial vascular repair, nasal packing); or postoperative retroperitoneal, intraspinal or intraocular bleeding (confirmed clinically or on imaging); or re-operation for surgical bleeding.

### Statistical analysis

SPSS software version 23.0 (IBM Corp., Armonk, NY, USA) was adopted to analyze the data. In all cases, a *P*-value of <0.05 was considered statistically significant. The sample size was calculated with G*Power 3 (http://www.gpower.hhu.de/). A sample size of 960 or more patients was required with a power of 0.8 and type I error of 0.05^26^. This was calculated under the assumption that the expected odds ratio (OR) for postoperative MACCE in patients who underwent noncardiac surgery with an interval from PCI to surgery of <one month compared to those with the interval ≥one month would be 1.5. The incidence of MACCE in patients with an interval from PCI to surgery ≥1 month was assumed to be 5%^8^. Sample size was also calculated for the multivariable logistic regression analysis according to the rule that outcome events should be ten per each independent predictor^27^. For this study, this was estimated to be 1148 patients or more to permit unbiased accommodation of seven or fewer predictive variables for the results of our multivariable logistic regression model (under the observed 6.1% incidence of postoperative MACCE)^27^.

Continuous variables were tested for their normal distribution by the Kolmogorov-Smirnov test and reported as median values with their interquartile ranges. Categorical variables were reported as absolute numbers (*n*) and relative frequencies (%). Missing data regarding baseline medical condition were present in <3% of records. There was no missing data regarding time from PCI, maintenance of antiplatelet agent, perioperative hematocrit values and amount of transfusion. Categorical variables were compared by the χ^2^ test or Fisher’s exact test according to their expected counts. Continuous variables were compared with the Student *t*-test and Mann-Whitney *U* test according to their distribution.

We conducted the following analyses according to our aims described in the introduction. Firstly, logistic regression models were used to identify predictors for MACCE within 30 days of surgery. Univariable logistic regression analysis was used first to identify possible risk factors for MACCE, with the multivariable model including all the variables included in the univariable analysis without any significance criterion. Continuous variables except for hemoglobin and albumin, as these variables had commonly used reference cut-offs, were not categorized before performing logistic regression analysis not to lose information. Predictor variables were selected from a list of candidate variables entered into the univariable analysis by performing a backward Wald stepwise variable selection with a significance criterion of *P* < 0.20. Potential variables included maintenance of antiplatelet agents and time from PCI to surgery.

**Figure 2:**
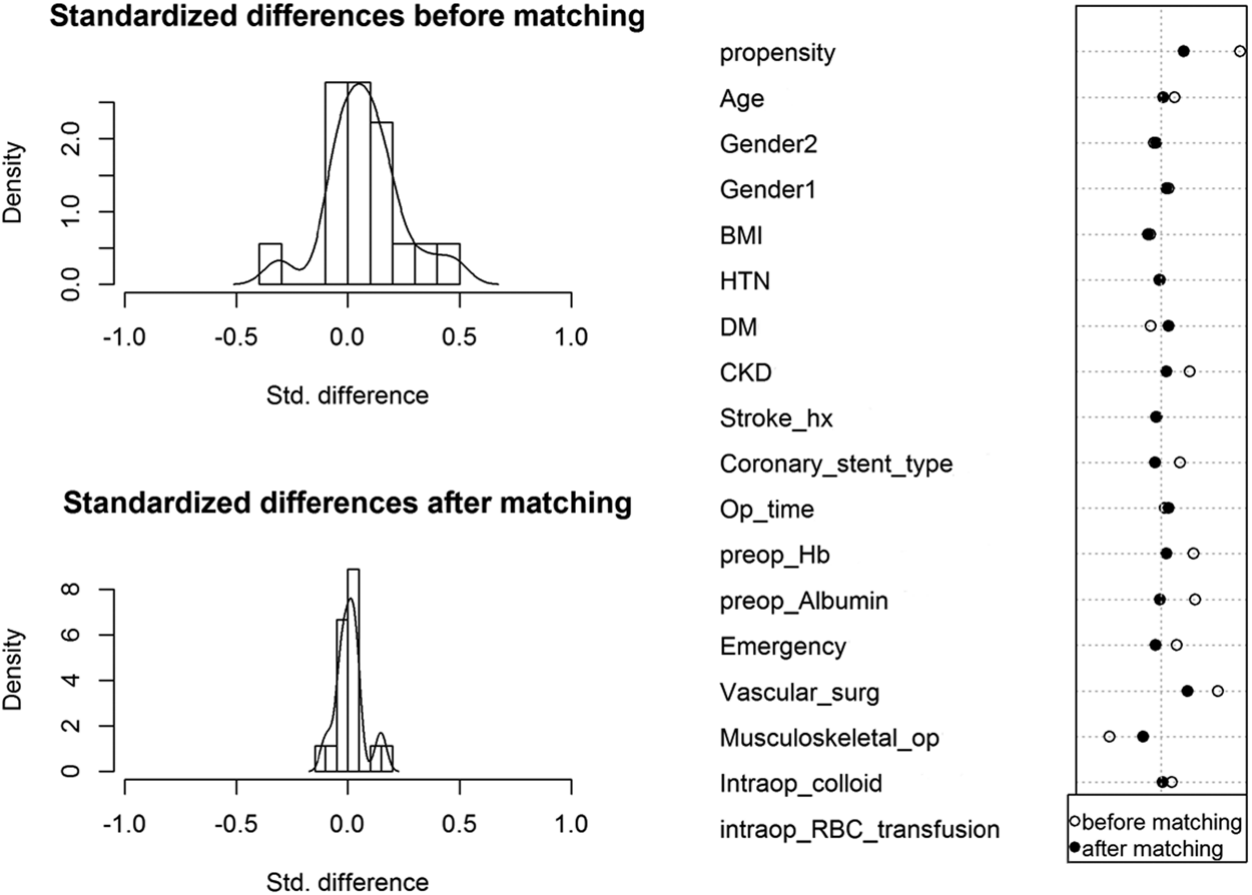
Histogram (left) and covariate balance plot (right) of distribution of standardized differences in the propensity scores between patients who maintained aspirin until surgery without cessation (n = 450) and those who did not maintain aspirin (n = 450, i.e. aspirin use before surgery but stopped ≥7 days before surgery or no use within 30 days before surgery). Values in the X-axis represent the percent standardized difference of covariates between the aspirin and non-aspirin groups. BMI = body-mass index, HTN = hypertension, DM = diabetes mellitus, CKD = choronic kidney disease, Op_time = operation time, preop_Hb = preoperative hemoglobin, intraop_RBC_transfusion = incidence of intraoperative red blood cells transfusion.

Secondly, we conducted a propensity score-matched analysis to match (1) patients who maintained and did not maintain aspirin before surgery, i.e. used before surgery but stopped ≥7 days before surgery or no use within 30 days before surgery and (2) those who underwent surgery before and after the time points including one month, six month, and one year. Binary logistic regression analysis was used to determine the probability of aspirin and non-aspirin group assignment, which was used for matching. We used a greedy method with 1:1 pair. A total of 450 patients who maintained aspirin was matched with those who did not maintain aspirin using the nearest neighbor matching (Fig. 2). A total of 235, 427, and 593 patients who underwent surgery before one, six, and twelve months after DES implantation were matched with those who underwent surgery after one, six, and twelve months (Supplementary Fig. S1). The following variables were used as a contributor to the propensity score: age, sex, body-mass index (BMI), history of hypertension, diabetes mellitus, chronic kidney disease, stroke, type of DES, surgery types, surgery time, emergency operation, preoperative hemoglobin and preoperative albumin. We defined the caliper as 0.2 standard deviations of the logit-transformed propensity score. The balance between the two groups was tested using standardized difference (>0.20 suggests imbalance). The incidence of MACCE, as well as the incidence of major bleeding, was compared in the matched cohort.

## Electronic supplementary material

Supplementary Table S1, S2, Supplementary Figure S1

Supplementary dataset
