# Supplementary material for: Risk of non-cardiac surgery after percutaneous coronary intervention with drug-eluting stents
Source: Sci Rep. 2017 Nov 27;7:16393. doi: 10.1038/s41598-017-16672-z (PMC5704017; doi:10.1038/s41598-017-16672-z)

Supplementary Information

Risk of non-cardiac surgery after percutaneous coronary intervention with drug-eluting stents

Sun-Kyung Park[1], **Dhong Eun Jung**[1], **Sung Ae Jung**[1], **Won Ho Kim**[1,*], **Jae-Hyon Bahk**[1]

[1] Department of Anesthesiology and Pain Medicine, Seoul National University Hospital, Seoul, Republic of Korea

Supplementary Table S1. Definition of the components of the primary study outcome. These definitions were modified from a previous study of Devereaux et al.[7]

Outcome	Definition
Myocardial infarction	Diagnosis of myocardial infarction required any one of the followings:<br>1. A typical rise of troponin or a typical fall of an elevated troponin detected at its peak post-surgery in a patient without a documented other explanation for the troponin change (e.g. pulmonary embolism) OR a rapid rise and fall of creatinine kinase-myocardial band (CK-MB). This finding should accompany one of the following:<br>a. ischemic sign or symptom<br>b. development of pathologic Q wave<br>c. electrocardiography (ECG) changes indicative of ischemia<br>d. coronary intervention (i.e., PCI or CABG surgery)<br>e. new or presumed new cardiac wall motion abnormality on echocardiography or new or presumed new fixed defect on radionuclide myocardial imaging<br>2. Development of new pathological Q wave on an ECG if troponin levels were not obtained or were obtained at times that could have missed the clinical event.
Non-fatal myocardial infarction	Non-fatal myocardial infarction was defined as successful patient treatment and resuscitation from either documented or presumed myocardial infarction.
Coronary revascularization	Cardiac revascularization procedure was defined as PCI or CABG surgery.

Pulmonary embolism	The diagnosis of pulmonary embolism required any one of the following:<br>1. Diagnosis suggested with a high probability by ventilation/perfusion lung scan<br>2. An intraluminal filling defect on pulmonary angiography<br>3. An intraluminal filling defect of segmental or larger pulmonary artery on a helical CT scan<br>4. A positive diagnostic test for deep vein thrombosis and one of the following<br>a. non-diagnostic ventilation/perfusion lung scan (i.e. low or intermediate probability suggested)<br>b. non-diagnostic helical CT scan (i.e. subsegmental defect or technically inadequate study)
Stroke	Stroke was defined as a new focal neurological deficit thought to be vascular in origin with signs or symptoms lasting more than 24 hours

CK-MB = creatinine kinase myocardial band, PCI = percutaneous coronary intervention, CABG = coronary artery bypass graft.

Supplementary Table S2. Definitions of components of secondary outcome of postoperative morbidity. Composite outcome of postoperative morbidity included MACCE as defined in Supplementary Table 1 and respiratory, cardiac, renal and other complications during postoperative 30 days.

Outcome	Definition
Cardiac complication	Cardiac complications included intra- and postoperative myocardial ischemia, postoperative newly onset arrhythmia such as atrial fibrillation, atrial flutter, ventricular tachycardia, pacemaker insertion, pericardial effusion and cardiac arrest.<br>An episode of myocardial ischemia was defined as ST depression or elevation in lead II, relative to the reference baseline electrocardiogram before induction of anesthesia, of 0.2 mV or more, lasting longer than 5 min. ST-segment analysis during surgery was performed by reviewing ST-segment values in the anesthesia record. Postoperative myocardial ischemia was determined according to the ICU charts or the 12-lead electrocardiogram checked postoperatively during hospital stay.
Respiratory complication	Respiratory complications included pleural effusion, pneumothorax, pneumonia, reintubation and pulmonary edema.
Renal complication	Renal complications included postoperative newly onset continuous renal replacement therapy, dialysis and acute kidney injury.
Other complication	Other complications included sepsis, reoperation due to postoperative surgical bleeding, and disseminated intravascular coagulopathy.

Supplementary Figure S1. Histogram (left) and covariate balance plot (right) of distribution of standardized differences in the propensity scores between patients who underwent surgery before one month after PCI with DES (n=235) and those with underwent surgery after one month since PCI (n=235). Values in the X-axis represent the percent standardized difference of covariates between the two surgery interval groups. BMI = body-mass index, HTN = hypertension, DM = diabetes mellitus, CKD = choronic kidney disease, Op_time = operation time, preop_Hb = preoperative hemoglobin, intraop_RBC_transfusion = incidence of intraoperative red blood cells transfusion.

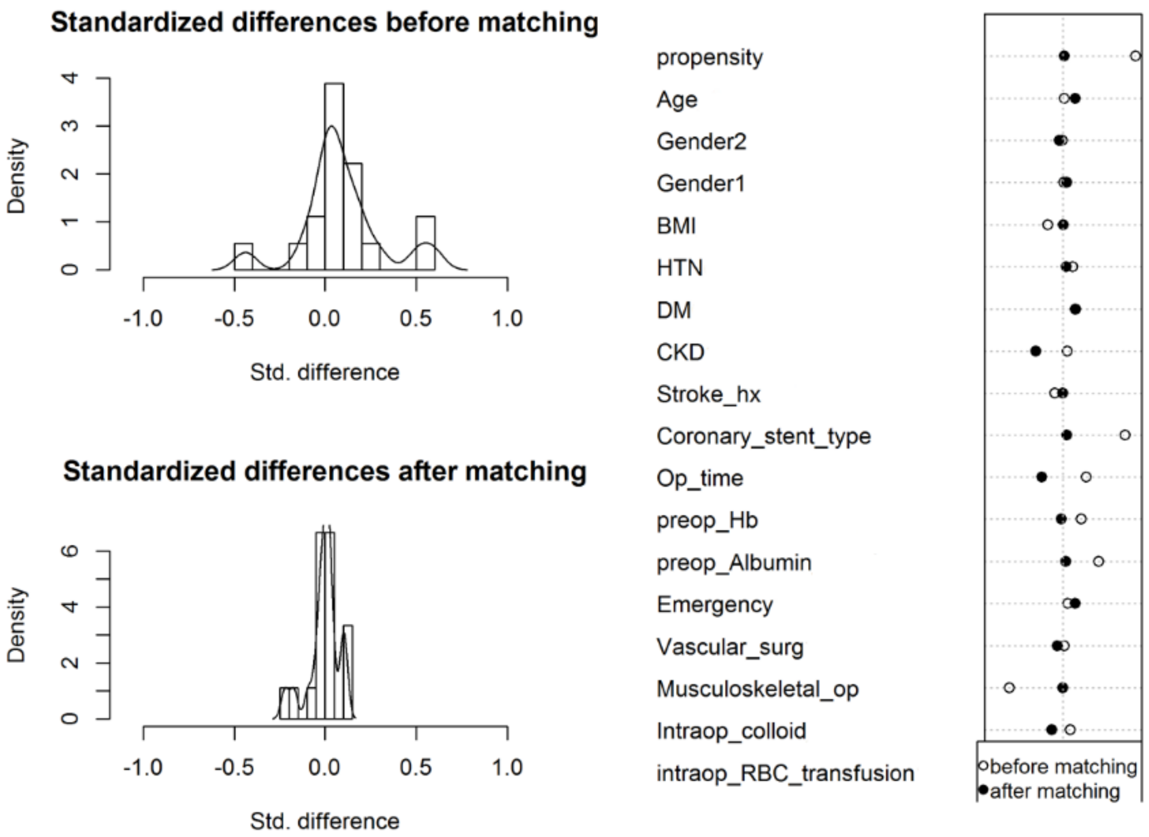

Supplement: Supplementary file 1 — Supplementary Table S1, S2, Supplementary Figure S1 [file 41598_2017_16672_MOESM1_ESM.pdf]
